# Exploring the Versatility of Transconjunctival Blepharoplasty: A Retrospective Single-Center Study

**DOI:** 10.7759/cureus.72929

**Published:** 2024-11-03

**Authors:** Rajat Gupta, Armaan Khosa, Priya Bansal, Gautam Chaudhury

**Affiliations:** 1 Plastic Surgery, Excel Hospital, New Delhi, IND; 2 Plastic Surgery, Adesh Medical College and Hospital, Shahbad, IND

**Keywords:** blepharoplasty, eyelids, orbital fat, tear trough deformity, transconjunctival

## Abstract

Background

Transconjunctival blepharoplasty using the postseptal technique provides direct access to orbital fat, offering reduced risk of complications, such as ectropion, entropion, fat over-resection, and inferior oblique muscle palsy. This study aims to evaluate the clinical outcomes and safety of transconjunctival blepharoplasty using the postseptal technique, focusing on the incidence of postoperative complications, the recovery time, and the feasibility of combining ancillary procedures with this approach.

Methods

In this retrospective observational study, we analyzed the outcomes of 88 patients who underwent transconjunctival postseptal blepharoplasty at a private hospital in northern India between July 2022 and January 2024. The patients, aged 20 to 76 years, received either general or local anesthesia. Follow-up assessments occurred at one, two, three, six, and 12 weeks postoperatively.

Results

The study population was composed of 28 men and 60 women, and the median age was 48 years. Of these patients, 31 underwent concurrent excess skin excision, 19 had additional canthoplasty or canthopexy, and 87 received fat transfer to correct a negative vector. Postoperative swelling, bruising, and chemosis were common, typically resolving within one to two weeks. Fourteen patients reported foreign body sensation, which resolved within 48 to 72 hours. The average time for the resumption of social activities was two weeks.

Conclusions

Transconjunctival blepharoplasty offers benefits such as reduced swelling, bruising, and risk of lid retraction, along with faster recovery, when compared to transcutaneous approach. Ancillary procedures can be safely combined with this approach. Further research is warranted to explore the long-term outcomes and efficacy of this technique across diverse patient populations.

## Introduction

The transconjunctival approach for lower eyelid blepharoplasty was first described in 1924 by French surgeon Bourguet [1].

The postseptal technique of transconjunctival blepharoplasty is a direct method of accessing the orbital fat without touching the septum. Indications for blepharoplasty through a transconjunctival approach are eye bags without excess skin, eye bags with skin excess where an incision for skin only is added, eye bags with lid laxity where a lateral canthal tightening procedure is added, and eye bags where it is necessary to treat the negative vector to achieve a smooth transition of the lid-cheek junction. The negative vector, which is caused by volume loss in the malar area and makes the malar eminence seem posterior to the corneal surface, can also be corrected by simultaneous fat grafting to the lid-cheek junction.

The transconjunctival approach avoids postoperative retraction (eyelid retraction or iatrogenic ectropion), as it avoids surgical intervention to the muscle and skin. [2] Complications such as ectropion, entropion, fat over-resection, and inferior oblique muscle palsy have been reported more frequently with the transcutaneous approach to blepharoplasty [3, 4].

We present the data of 88 patients who underwent transconjunctival blepharoplasty with the postseptal technique. This article elaborates on the technique and records the operative time and postoperative follow-up.

## Materials and methods

This retrospective observational study was conducted at a private hospital in northern India. A total of 88 patients were studied over a period of 18 months, from July 2022 to January 2024. Participants were between the ages of 20 and 76 years.

All adult patients who underwent transconjunctival post-septal blepharoplasty, under general or local anesthesia, have been included in the study. All patients with a history of systemic disease, previous eyelid surgery, a history of eye trauma, and with symptoms of severe dry eye syndrome were excluded from the study.

A preoperative assessment was conducted, which included a thorough medical and surgical history. The periorbital region was examined, including the eyelid position; skin, muscle, and lateral canthal tendon laxity; condition of the skin; prolapse of orbital fat, and volume loss in the cheek. Dry eyes were ruled out. Blood tests, including a complete blood count, renal and liver function tests, bleeding and clotting profiles, fasting blood sugars, and viral markers, were conducted, along with a chest X-ray and an electrocardiogram for all patients to confirm fitness for surgery.

Patients were called for admission on the day of surgery. Written informed consent for the surgery and medical photography was obtained from each patient. The study was approved by the Genebandhu Independent Ethics Committee (ECG027/2024) and was conducted in accordance with the principles of the Declaration of Helsinki. Photographic documentation was performed during the preoperative and postoperative periods. We followed up with all patients at one week, two weeks, three weeks, six weeks, and 12 weeks after surgery.

Procedure

**Figure 1:**
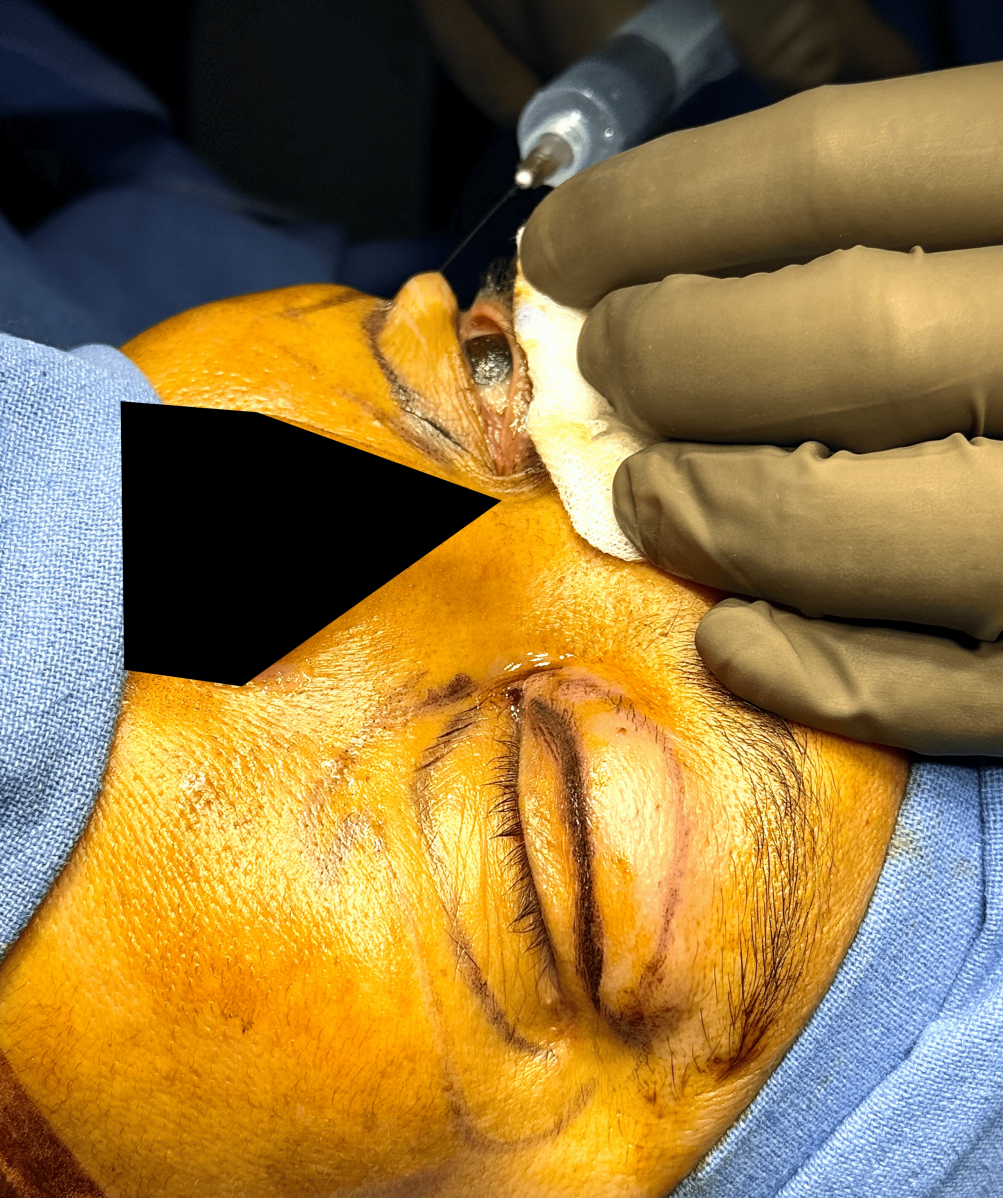
Infiltration of anesthesia into the lower eyelid skin.

With the patient in a supine position and under aseptic precautions, cleaning and draping were performed. Local anesthesia was infiltrated into the lower eyelid and the inferior orbital rim (Figure 1).

**Figure 2:**
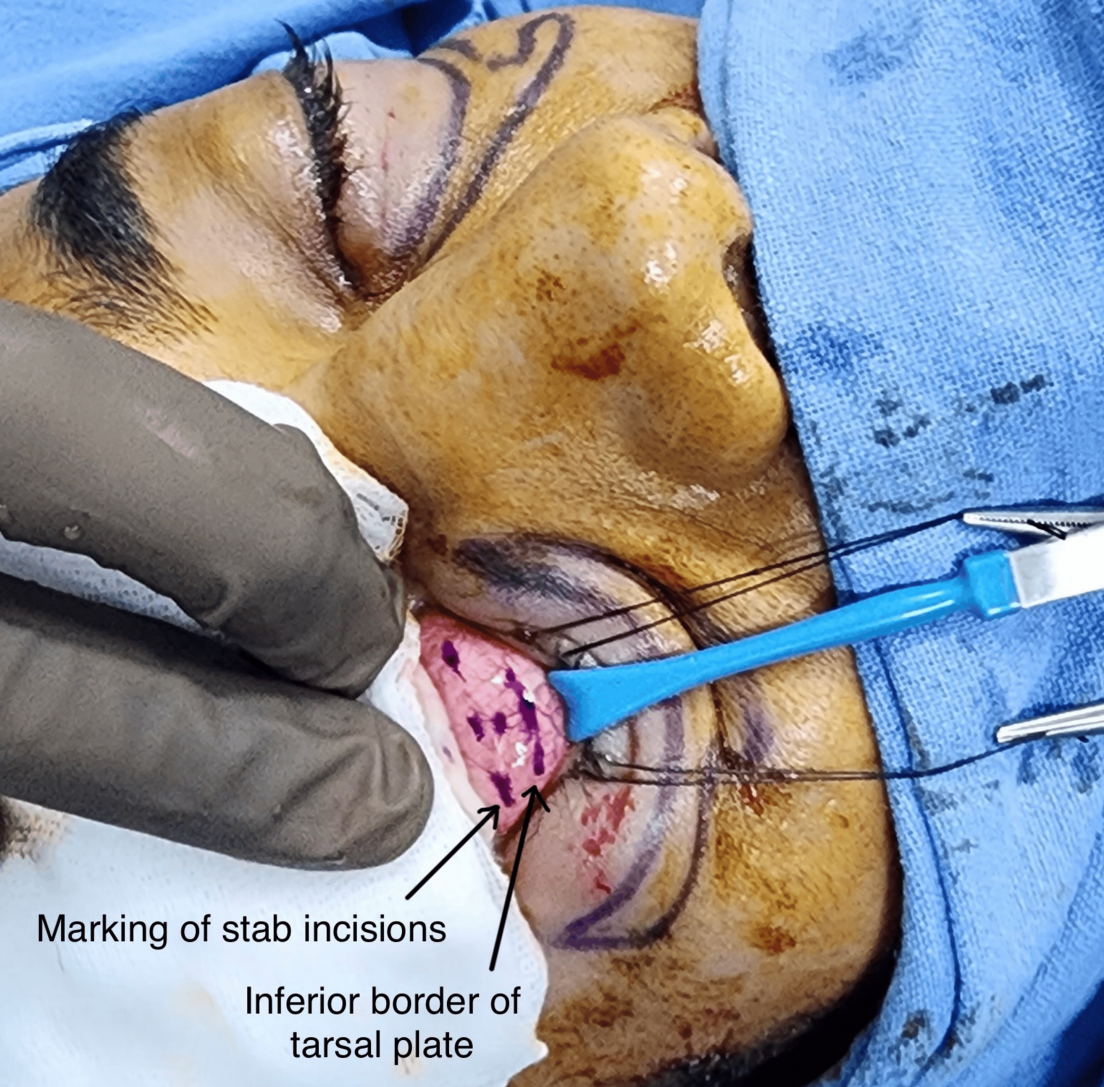
Marking of inferior border of tarsal plate of lower eyelid and the marking for stab incisions for transconjunctival blepharoplasty.

A cotton pad soaked in saline was applied as a corneal shield over the eyeball, and the lower eyelid was retracted with a Desmarres retractor. Separate stab incisions, measuring 3 mm each, were taken for the three orbital fat compartments, all of which were around 5-6 mm below the lower margin of the tarsal plate, avoiding the inferior conjunctival fornix (Figure 2).

**Figure 3:**
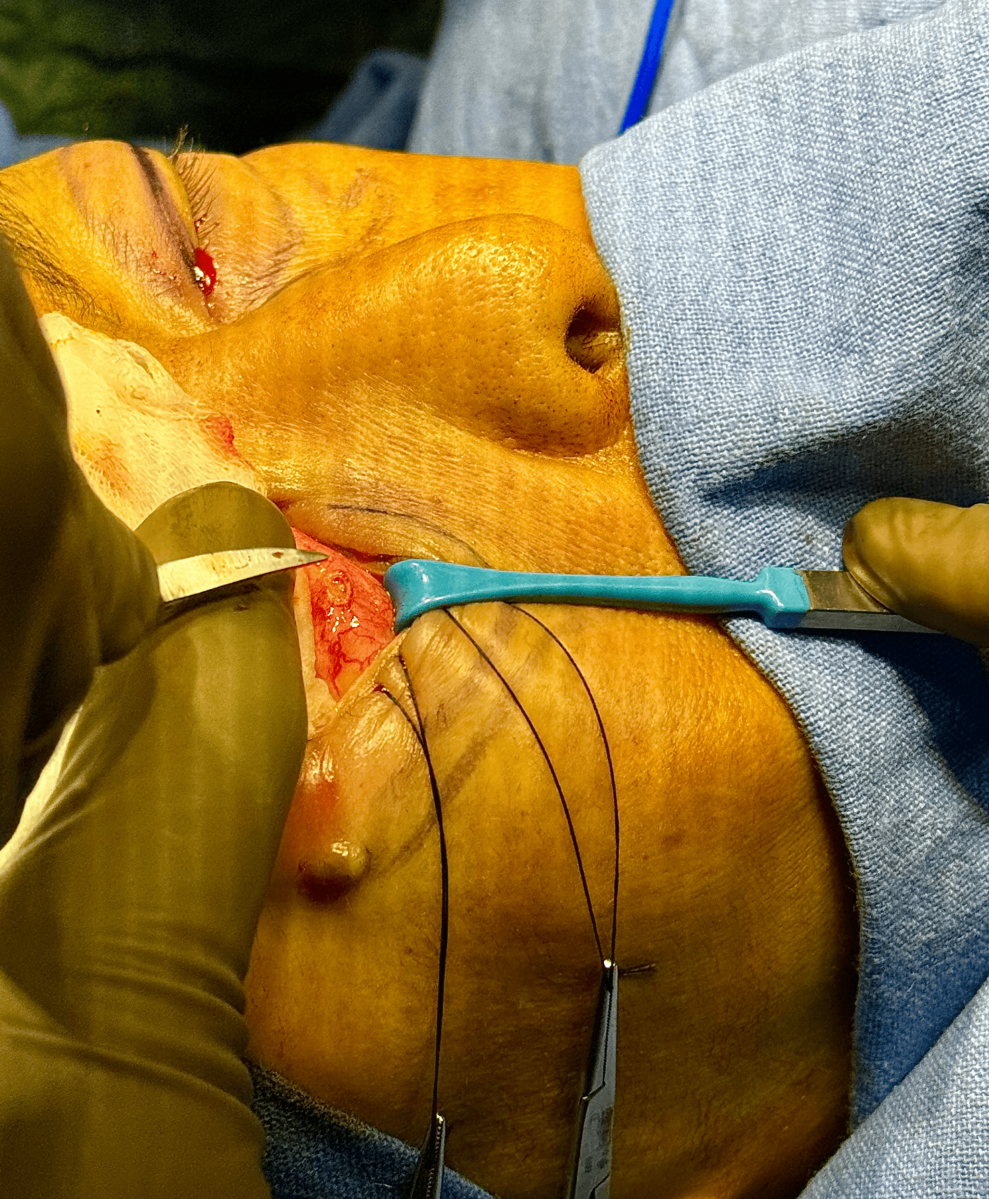
The stab incision. The stab incision measures 3 mm in transconjunctival postseptal approach of lower eyelid blepharoplasty. The instrument is pointing to the stab incision.

The central compartment stab incision was taken first (Figure 3).

**Figure 4:**
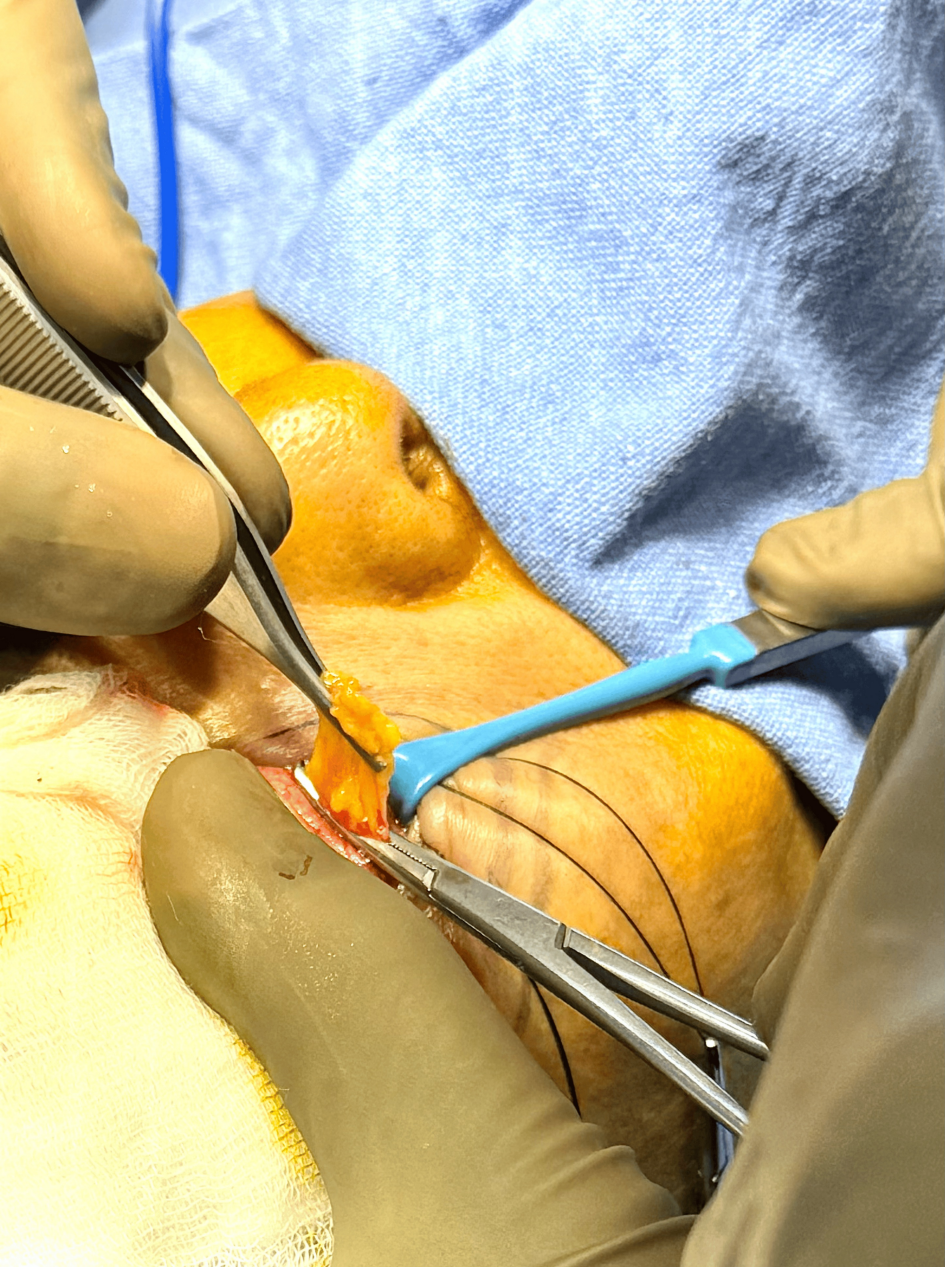
The infraorbital fat popping out and being held at the base with a hemostat.

The stab incision was opened with a hemostat. Gentle pressure on the globe was applied until the intraorbital fat appeared. The fat was gently grasped with forceps, and local anesthesia was infiltrated into the fat. The prolapsed fat was held, and a hemostat was placed at its base (Figure 4). It was then cauterized and removed.

**Figure 5:**
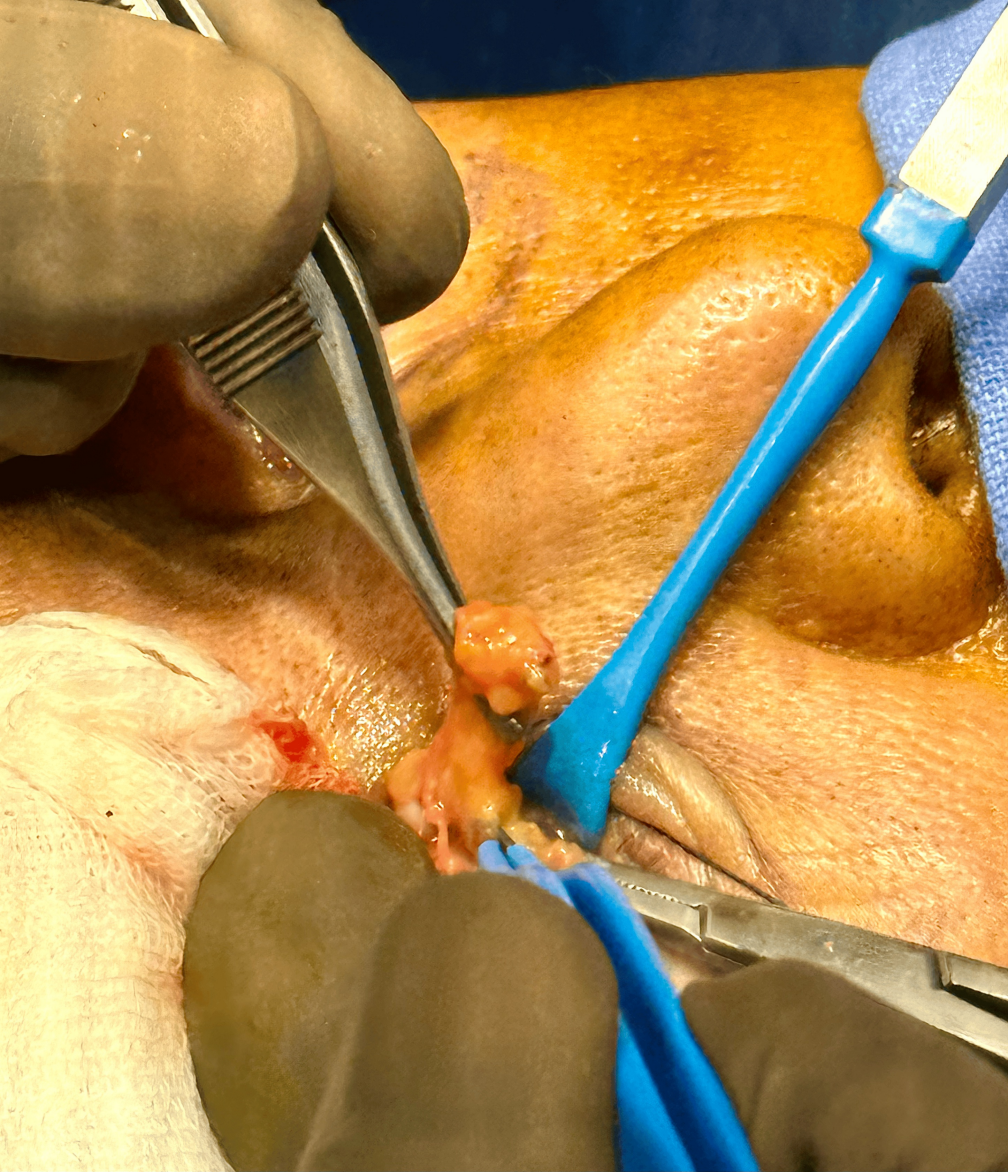
Fat being cauterized.

Radiofrequency cautery was used to treat the visible remaining fat (Figure 5). This step enables the remaining fat to shrink. We ensured that some residual fat was left behind to avoid the appearance of hollowness. Similar steps were performed for the lateral and nasal fat compartments. Stab incisions had a distance of at least 8 mm between them. Since the incisions were very small, they healed well within 48 hours and did not require any sutures.

**Figure 6:**
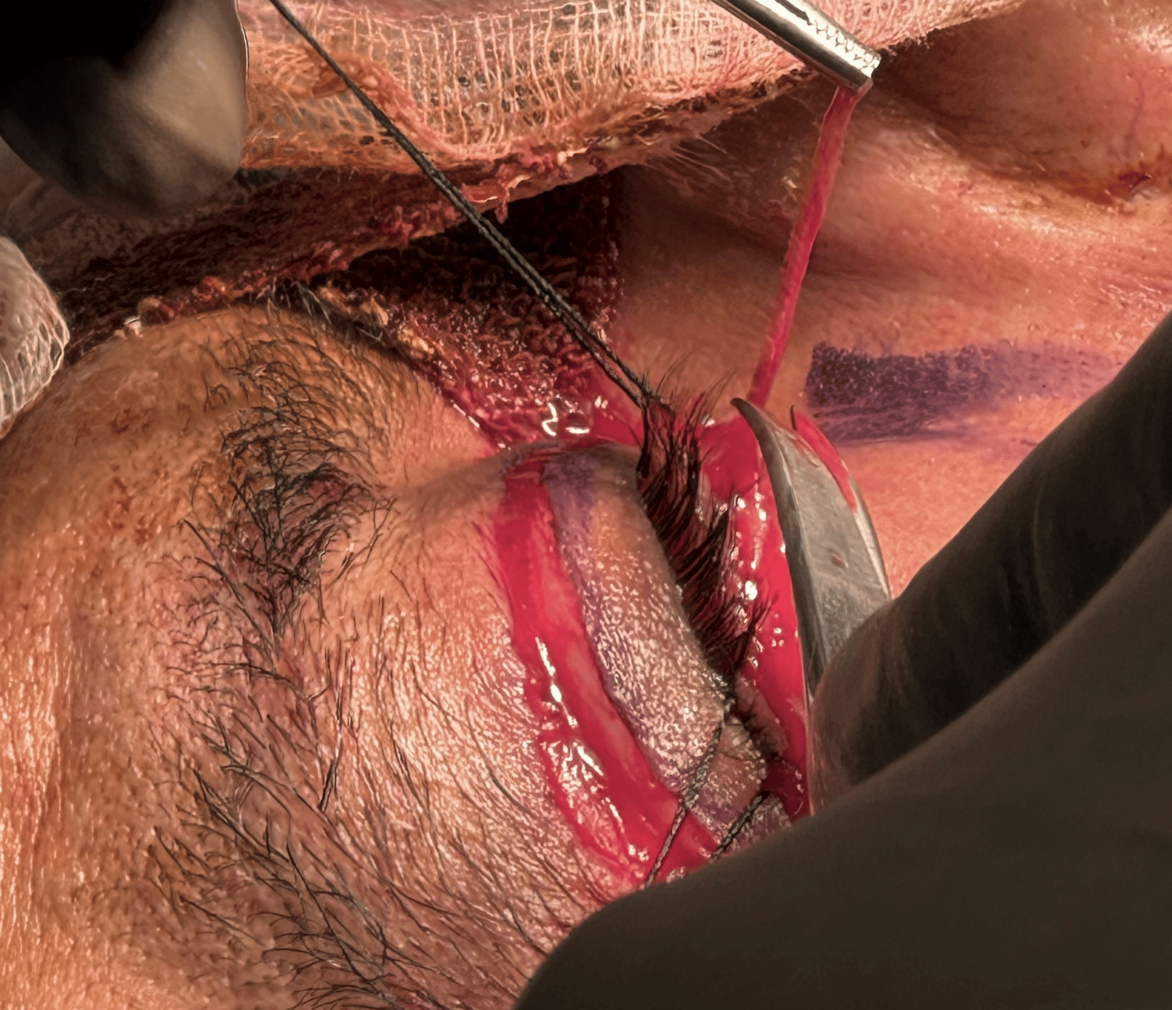
The thin skin strip being removed.

In patients who had excess skin requiring removal, a subciliary incision was made after the transconjunctival blepharoplasty, and the skin strip was excised (Figure 6).

**Figure 7:**
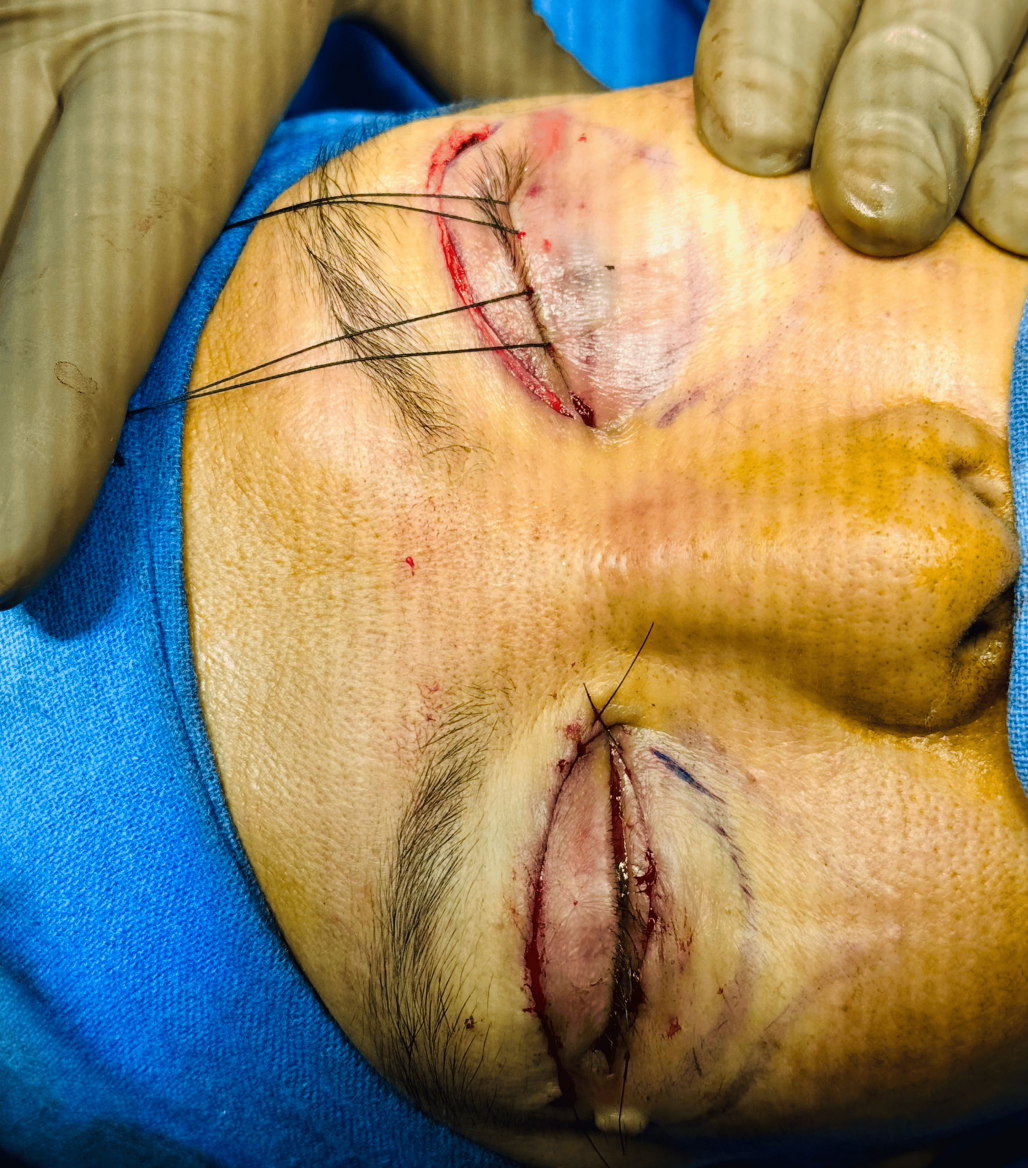
Immediate postoperative result. The figure shows the fine closure of the incision for skin strip removal in the right eye and after transconjunctival blepharoplasty in both eyes.

The skin was closed with a subcuticular 5-0 reverse-cutting non-absorbable suture (Figure 7).

In most cases, patients also needed correction of the tear trough deformity, which requires volume addition to the suborbicularis oculi fat (SOOF) and deep medial cheek fat compartment to enhance the youthful appearance. This was achieved with microfat grafting.

We did not transpose the orbital fat into the SOOF region, which involves releasing the orbicularis retaining ligament (ORL). We believe that releasing this important structure creates permanent communication between the SOOF fat compartment and the lower eyelid. Any intervention, such as injecting fillers or fat transfer to the SOOF compartment later in life, can cause migration of fat or filler in the lower eyelid, mimicking the reappearance of the eye bags.

Post-operative instructions

We discharged all patients on the same day after a six-hour observation period.

We advised ice fomentation around the eyes for the first 48 hours. Ophthalmic antibiotic eye ointment was given for use twice a day. Artificial tears were used twice or three times a day. Oral analgesics were added. We advised patients to use a pillow for head-end elevation.

Skin sutures, when present, were removed on the fifth postoperative day.

We called all 88 patients to OPD for a follow-up at one week, two weeks, three weeks, six weeks, and 12 weeks after surgery.

## Results

We studied a total of 88 patients, of whom 28 were male and 60 were female. All patients were between 20 and 76 years old, with the most common age group being 40-50 years old.

Ancillary procedures were added to transconjunctival blepharoplasty in several cases. Thirty-one patients (35.23%) had excess skin excision along with transconjunctival blepharoplasty. Nineteen patients (21.59%) underwent canthoplasty or canthopexy. Eighty-seven patients (98.86%) had fat transfer to correct the negative vector.

The total operative time was recorded for the procedure. It included lower transconjunctival blepharoplasty performed alone, combined with skin excision, combined with canthopexy or canthoplasty, and combined with upper blepharoplasty in the same sitting. The average operative time was taken when lower transconjunctival blepharoplasty was performed alone or when it was combined with other procedures, and the results were recorded (Table 1).

**Table 1:** Average operative time for lower transconjunctival blepharoplasty when done alone and when combined with other procedures.

Procedure	Total average operative time
Lower transconjunctival blepharoplasty	8 minutes 47 seconds
Lower transconjunctival blepharoplasty with skin excision	11 minutes 9 seconds
Lower transconjunctival blepharoplasty with lateral canthoplasty/ canthopexy	20 minutes 37 seconds
Lower transconjunctival blepharoplasty with upper blepharoplasty	25 minutes 22 seconds

All patients had swelling, bruising, and some degree of chemosis after the surgery. Most symptoms resolved between one and two weeks. Fourteen patients (15.73%) experienced some foreign body sensation, which resolved between 48 and 72 hours. Nine patients experienced a resolution of symptoms within 48 hours and five within 72 hours. Follow-up was recorded at the end of one, two, three, six, and 12 weeks in the OPD (Table 2).

**Table 2:** the postoperative follow-up results for all patients at one, two, three, six, and 12 weeks. Postoperative follow-up results for all patients at one, two, three, six, and 12 weeks for swelling, bruising, chemosis, foreign body sensation and symptomatic dry eyes.

Sequelae	1 week	2 weeks	3 weeks	6 weeks	12 weeks
Swelling	69(78.41%)	11(12.5%)	2(2.27%)	0	0
Bruising	77(87.5%)	9(10.23%)	1(1.14%)	0	0
Chemosis	10(11.36%)	0	0	0	0
Foreign body sensation	0	0	0	0	0
Symptomatic dry eye	19(21.59%)	17(19.32%)	2(2.27%)	2(2.27%)	0

The time taken by patients to get back to social life was also recorded, with most patients taking two weeks (Table 3).

**Table 3:** Time taken by patients to get back to social life

Time taken to resume social life	Percentage of patients
1 week	22%
2 weeks	67%
3 weeks	9%
4 weeks	2%

There were no complications, including hemorrhage, visual problems, injury to the inferior oblique muscle, or corneal trauma. No long-term complications, such as lid retraction or dry eye, were noted.

**Figure 8:**
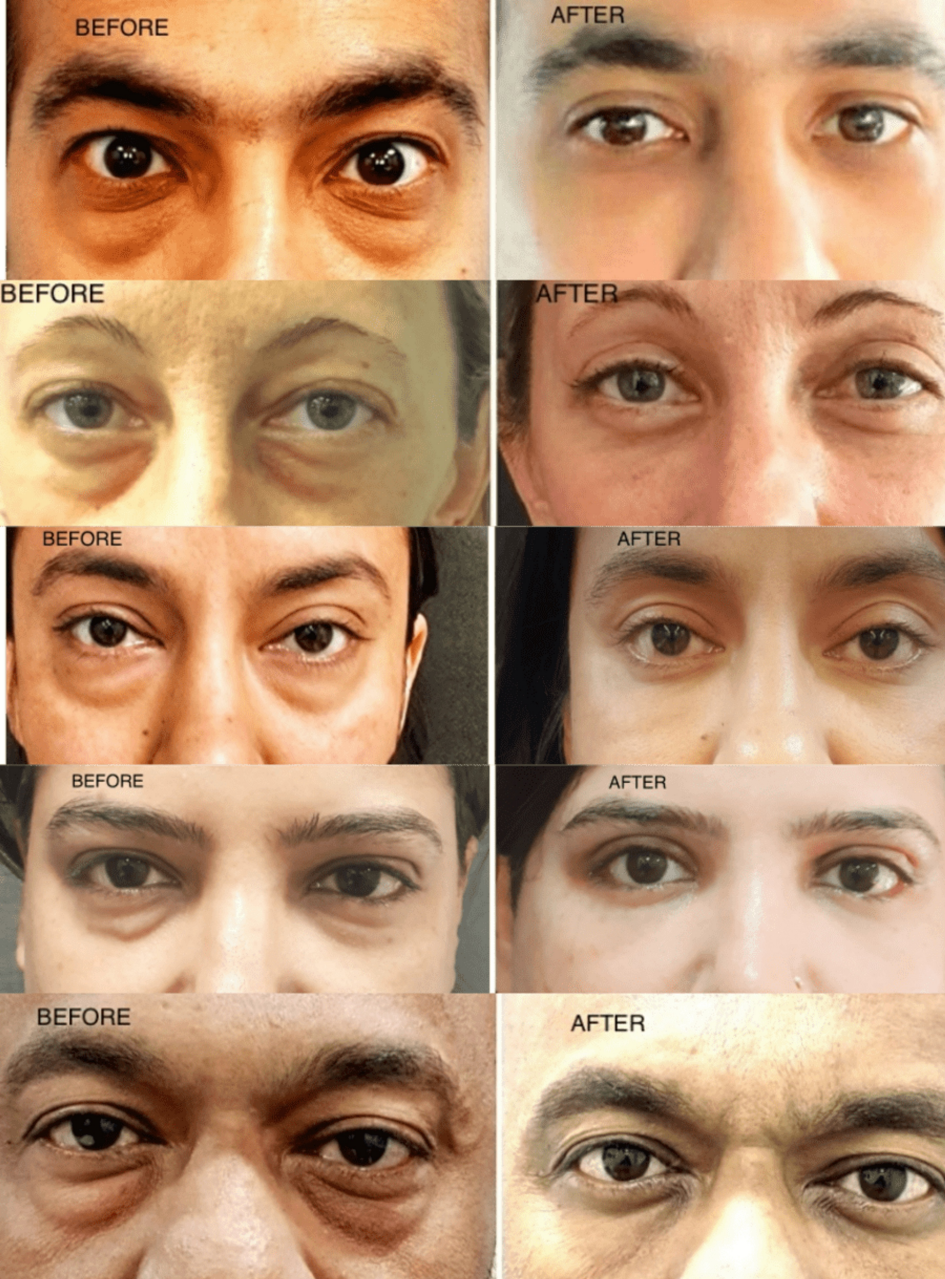
Preoperative images and postoperative images at four weeks after transconjunctival blepharoplasty.

Preoperative and postoperative pictures of a few of our patients with lower transconjunctival blepharoplasty with the post-septal method are attached. The postoperative results are at the end of four weeks (Figure 8).

## Discussion

The periorbital region experiences significant changes during the aging process. The physiological changes include laxity of the skin, orbital septum, canthal tendons, and orbicularis oculi muscle [5]. These changes manifest as orbital fat prolapse, the development of malar festoons, crow’s feet-like radiation, and wrinkles around the eyes [6].

Lower eyelid blepharoplasty can be performed via two approaches, namely the transconjunctival and the transcutaneous (subciliary) approaches. The transconjunctival approach has a faster learning curve and a lower operative time. It leaves no scars and has minimal chances of postoperative skin retraction [5].

Anatomically, the lower eyelid is subdivided into three lamellae: (a) an anterior lamella, which includes the skin and orbicularis oculi muscle; (b) a middle lamella, comprising the orbital septum; and (c) a posterior lamella, composed of the tarsal plate, eyelid retractors, and palpebral conjunctiva [7, 8]. The septum separates the orbital fat from the overlying structures. There are three retroseptal fat pads: the medial, central, and temporal. The inferior oblique muscle lies between the central and medial fat pads, predisposing it to intraoperative injury [9].

Lower eyelid blepharoplasty aims to reduce the prominence of the lower lid-cheek junction and recreate a smooth transition at the lower lid-cheek interface [5]. The prolapse of orbital fat in the lower eyelids, referred to as steatoblepharon, commonly causes the appearance of “bags under the eyes” [10] and is graded as follows:

S = -1: Very prominent fat pads

S = 0: Mildly noticeable fat pads

S = +1: Absence of all three fat pads

S = +2: Hollowing of periorbital fat

The common indications of lower eyelid blepharoplasty incorporate rhytidosis and dermatochalasis, relative steatoblepharon, tear trough deformity, infraorbital collapse, malar festoons, and lower lid asymmetry. Many of these are related to a double convex deformity of the lower eyelid [5]. The transconjunctival approach is best suited for patients with steatoblepharon with minimal to moderate lower lid laxity [11]. Contraindications for the transconjunctival approach include active blepharitis, undetected dry eye syndrome, a prior history of refractive procedures (surgery should be postponed for at least six months), smoking, and anticoagulants (these should be withheld for at least two weeks before surgery [9, 12-14].

Transconjunctival blepharoplasty can be performed in two ways: (a) preseptal, which requires septal division to access fat; and (b) postseptal, which is a direct approach to fat with preservation of the orbital septum [8].

An American survey suggested that 80% of surgeons perform fat repositioning in lower eyelid blepharoplasty. Approximately 17% prefer supraperiosteal fat relocation because it is a faster and technically simpler procedure [15]. The senior author of the present study has experience in fat repositioning, where a defect in ORL and a flap of fat is made and sutured to the periosteum in the SOOF compartment. We believe that permanently releasing this important structure (ORL) creates communication between the SOOF fat compartment and the lower eyelid. The senior author noticed in the long-term follow-up of these patients that when they need reaugmentation of SOOF with dermal fillers, this communication between SOOF and eyelids could cause the reappearance of the eye bags when filler was injected later into the suborbicularis oculi fat region. The release of the ORL during fat repositioning is believed to be the cause of this phenomenon. Eye bags that reappeared after injecting dermal fillers were successfully treated with a hyaluronidase injection, thus confirming the hypothesis. We thereafter stopped releasing the ORL ligament and now only use microfat deposits to augment the SOOF compartment.

Fractionated fat has been injected both above the periosteum and below the muscle in the infraorbital rim region as an additional step in lower blepharoplasty by Rohrich et al., further blending the eyelid-cheek junction [16].

Complications associated with blepharoplasty within the first postoperative week include corneal abrasions and retrobulbar hemorrhage, the latter threatening vision. In the intermediate period, between weeks one and six, there can be upper and lower eyelid positional problems, including ptosis or lagophthalmos, strabismus, epiphora, and corneal exposure [17]. The late complications, which occur later than six weeks, include changes in eyelid height and contour, asymmetries, scars, and persistent edema [17]. All our cases of symptomatic dry eyes gradually improved by 12 weeks with lubrication. Swelling, bruising, and chemosis are normal sequelae of blepharoplasty. There can also be some degree of foreign body sensation.

The limitation of this study is that the authors have not made a comparative study with transcutaneous blepharoplasty. A comparative study of transcutaneous and transconjunctival approaches, with one of the methods being used in one of the lower eyelids of each patient, with a larger sample size, could be conducted in the future to give a definitive opinion on the indications and outcomes of the two techniques. This could provide a clear picture of how muscle involvement in the transcutaneous approach affects the recovery from surgery.

## Conclusions

Transconjunctival blepharoplasty offers the advantages of less swelling, less bruising, fewer chances of lid retraction, and faster recovery as compared to transcutaneous blepharoplasty. Initially, transconjunctival blepharoplasty was thought to be for younger patients, but today, with the advent of the skin pinch technique and the addition of canthopexy or canthoplasty, it is known that ancillary procedures can be safely added to transconjunctival blepharoplasty. This has widened the spectrum of indications for transconjunctival blepharoplasty. We believe that our experience can be helpful for oculoplastic and plastic surgeons willing to expand their armamentarium with versatile transconjunctival blepharoplasty.
